# A Comparative Survey of Feature Extraction and Machine Learning Methods in Diverse Acoustic Environments

**DOI:** 10.3390/s21041274

**Published:** 2021-02-11

**Authors:** Daniel Bonet-Solà, Rosa Ma Alsina-Pagès

**Affiliations:** Grup de Recerca en Tecnologies Mèdia (GTM), La Salle—URL, c/Quatre Camins, 30, 08022 Barcelona, Spain; daniel.bonet@salle.url.edu

**Keywords:** acoustic sensor, acoustic event detection, corpora, feature extraction, machine learning

## Abstract

Acoustic event detection and analysis has been widely developed in the last few years for its valuable application in monitoring elderly or dependant people, for surveillance issues, for multimedia retrieval, or even for biodiversity metrics in natural environments. For this purpose, sound source identification is a key issue to give a smart technological answer to all the aforementioned applications. Diverse types of sounds and variate environments, together with a number of challenges in terms of application, widen the choice of artificial intelligence algorithm proposal. This paper presents a comparative study on combining several feature extraction algorithms (Mel Frequency Cepstrum Coefficients (MFCC), Gammatone Cepstrum Coefficients (GTCC), and Narrow Band (NB)) with a group of machine learning algorithms (*k*-Nearest Neighbor (kNN), Neural Networks (NN), and Gaussian Mixture Model (GMM)), tested over five different acoustic environments. This work has the goal of detailing a best practice method and evaluate the reliability of this general-purpose algorithm for all the classes. Preliminary results show that most of the combinations of feature extraction and machine learning present acceptable results in most of the described corpora. Nevertheless, there is a combination that outperforms the others: the use of GTCC together with kNN, and its results are further analyzed for all the corpora.

## 1. Introduction

Years after the first surveillance systems, mainly based on camera networks [1], privacy issues started to arise [2], and, for many applications, the change from image detection in indoor or outdoor spaces tended to move to acoustic sensor networks [3], less intrusive for the life of citizens and with a wide spectrum of possibilities in terms of identification of events. Audio event detection (AED) and classification is of the upmost importance in several environments and applications. Identification of certain indoor sounds has been useful in several surveillance contexts, especially those related to human activity [4]. It helps in monitoring old or dependant people at home and triggering an alarm when some specific event is detected. Unobtrusive AED in smart homes has direct applications in ambient assisted living [5]. Identifying sounds related to breaking into houses or violent acts associated to crime or even terrorism has obvious security applications [6].

Any acoustic event detection algorithm previously analyzed and evolved in the laboratory can today be easily deployed in a real-time signal processing Wireless Acoustic Sensor Network (WASN) [3], due to technological advances of Internet of Things (IoT) in the framework (mainly) of the smartcities. These advances in sensor networks have also reached home environments, especially focusing in Ambient Assisted Living (AAL) applications [7], with appealing challenges as providing users of an AAL environment aimed to detect domestic accidents (e.g., falls, flooding, fire), or monitor their health by means of the behavioural analysis, with the final pretension of improving their quality of life [8]. Projects, such as CIRDO [9], focus on the safety of elderly and dependant people at home. Another project, homeSound [10], presented an AAL platform able to detect specific audio events in real-time enabling remotely tracking of a patient status by the medical staff using a decentralized intelligence architecture.

Detecting sounds in outdoor environments has also several applications ranging from traffic noise mapping in a city [11] to soundscapes modeling [12] or open air surveillance [13]. The DYNAMAP project [14], for instance, distributed a low-cost WASN in Rome and Milan in order to monitor a very specific environmental noise, i.e., Road Traffic Noise (RTN), and evaluate its impact in urban and suburban areas. The SONYC project (Sounds of New York City) [15] monitors the urban soundscape of New York City through a network of low-cost sensors. Characterizing and identifying acoustic scenes or soundscapes have also multiple uses, such as context-aware user experiences [16] or even in robot navigation [17], where it can augment visually obtained information. AED in surveillance systems can provide additional information about an incident or a dangerous situation and can be used along with security cameras and CCTV circuits [18]. Finally, Biology is also a field where these technological advances may be applied to evaluate the effects of any human activity to the well-being of animals [19]. Bioacoustics has been studying the acoustic monitoring of birds through the application of sound recognition techniques, following both manual or semi-automatic approaches [20]. Even wildlife monitoring can benefit from AED, by tracking endangered species [21] or by helping in species recognition tasks [22].

The AED process requires extracting acoustic features out of the data available and feeding them into a machine learning (ML) algorithm responsible for the classification. Multiple combinations of feature extraction (FE) methods and ML algorithms have been used in recent years on a wide range of scenarios. Mel Frequency Cepstrum Coefficients (MFCC) [23] are often used as a baseline in addition to more sophisticated FE methods which aim to improve the outcome of the AED applications. Gammatone Cepstrum Coefficients (GTCC) [24] has repeatedly achieved better identification accuracy than MFCC both in indoor and outdoor environments. In the particular case of soundscapes modeling, extracting Auto-Correlation Features (ACF) has been successfully used in Reference [25]. While classical ML algorithms, such as *k*-Nearest Neighbor (kNN), Support Vector Machines (SVM), and Gaussian Mixture Model (GMM), offer viable solutions [26], the use of algorithms based on Neural Networks (NN) is becoming one of the preferred choices present in many recent studies [27].

This paper aimed to study multiple combinations of classical ML algorithms with FE methods. The goal was to use some of the most relevant FE methods, which include MFCC, GTCC, and Narrow Band Auto-Correlation Features (NB-ACF), in addition to several ML algorithms (kNN, NN, and GMM), to apply to different environments and corpora with the final goal of obtaining the most flexible classical acoustic event detection algorithm to be deployed in any low-cost acoustic sensor platform. Among the audio data chosen, there are indoor, outdoor, soundscapes, surveillance-related, and birds sounds distributed in five multi-class datasets. One of the main objectives was to spot the strengths and weaknesses of each FE/ML pairing while assessing their performance according to different kinds of sounds and corpora. Related literature often opts for a vertical approach centering on a particular set of data. Results vary according to the kind of sounds and the time window chosen when performing its detection and classification, making an across-the-board comparison difficult. The goal of this paper was to offer a more general approach to evaluate the performance comparison of all the implemented algorithms over the proposed scenarios. It is known from previous works that the training and test of machine learning algorithms varies substantially [28] when going to the simulations in the laboratory with a small corpus into real-operation environment, where larger corpora are used [29], and several unpredictable events can occur. This work faced only the first part of the tests, near a proof of concept of the best combination of FE and ML for each type of group of sounds.

This paper is structured as follows. Section 2 describes recent related work in the topic, specifically research on sound events recognition. Section 3 contains an accurate description of the five datasets used. In Section 4, a brief description of the chosen FE and ML algorithms is provided, and, in Section 5, the overall results are presented, paying special attention to the best performing setup. Finally, Section 6 presents the conclusions of this work.

## 2. Related Work

In this section, we summarize some of the most relevant recent research on audio recognition and events detection in different environments, paying special attention to the ones that share the same FE methods and ML techniques chosen in this paper. As the number of studies on audio recognition is very extensive, only those that work with datasets similar to the ones in this project have been taken into account.

### 2.1. Acoustic Event Detection Algorithms

AED is typically achieved by two sequentially performed stages consisting in a process of FE followed by a ML algorithm. Firstly, the FE method provides a feature vector for each portion of the chosen audio. This vector is a highly compacted representation of the given signal, which is imperative to reduce the computational cost of the following step. Secondly, the ML algorithm is trained with this representative data in order to create a model of the sounds of interest which, in turn, will be used to predict future apparitions of those.

#### 2.1.1. Audio Features Extraction Methods and Its Applications

MFCC is probably one of the most broadly used methods in audio recognition, not only for speech [30] but also for a wide range of different sounds like surveillance-related events [31], soundscapes [32], or even animal sounds [22]. There is abundant literature related to events and anomalies detection in an outdoor environment. In Reference [6], the authors choose MFCC, among others, in order to extract the features out of a dataset of surveillance-related sounds obtained under pseudo-real-world conditions. In Reference [31], three types of abnormal sounds are detected: gun-shots, broken glasses, and screams, previously mixed with background sound successfully using a set of audio features which includes MFCC. Shifting to a multi-class event detection scenario, there are also several studies that have opted for MFCC as a way of obtaining meaningful features, such as Reference [32], where a dataset of 61 classes mixed with ambient background noise is used to analyze ten different soundscapes: basketball, beach, bus, car, hallway, office, restaurant, shop, stadium, and street. Another example can be found in Reference [33], where ten different classes of real-life urban sounds are classified. The dataset groups the classes in five main categories: community, construction, emergency, mechanical, and traffic. In this research, and many others, MFCC is chosen since it is a competitive baseline to benchmark novelty audio event detection methods.

MFCC has been repeatedly used in studies about urban and road traffic environments. In Reference [34], authors implement MFCC alongside MPEG-7 Low-Level Descriptors (LLD) and Perceptual Wavelet Packets (PWP) while studying nine urban traffic audio classes with samples obtained from professional sound effects collections including the detection of crashes. Moreover, in References [11,26], Socoró et al. come up with a thorough research on anomalous noise events within road traffic noise that also considers MFCC to parameterize the audio data.

This general use as a baseline method for features extraction is not restricted to outdoor and soundscape datasets. MFCC is chosen to perform a comparative between several classifying algorithms in Reference [35], using different datasets, including both indoor and outdoor sounds. Authors highlight its favorable properties at computing distances between different sounds. Other examples of indoor use of MFCC would be [4,10]. In the former, this technique is used in the design of a platform that detects audio events happening in daily life environments to help tracking the status of a patient in an Ambient Assisted Living (AAL) situation, while, in the latter, MFCC are used in combination with several other features to classify five classes of indoor audio events using six different machine learning algorithms.

Finally, MFCC has appeared multiple times in recent studies on bird songs recognition [21,36], where the songs’ fingerprints of some endangered species were successfully detected. In addition, in Reference [22], Somervuo et al. parameterize 14 common North-European bird species, and, in Reference [37], 40 different birds are identified. In both cases, MFCC is being used. More recently, MFCC has been used as a part of a two-stage approach to detect and classify woodpecker sounds [38].

Even if MFCC is probably the most widely spread, there are other feature extraction techniques that are being used in AED and sounds recognition. GTCC, for instance, has been chosen lately in several AED studies showing improvements in the accuracy of the detection when combined with different machine learning algorithms. In Reference [11], GTCC was extracted for the purpose of detecting anomalous noise events in a road traffic environment in addition to MFCC. Both coefficients (MFCC and GTCC) are also chosen in other studies about environmental sound and acoustic events classification. On the one hand, in Reference [24], two distinctly different datasets with several environmental sounds are classified: ESC-50 and UrbanSound8K, and a modified Gammatone filterbank is proposed. On the other hand, in Reference [39], two other publicly available datasets containing, respectively, 12 and 16 indoor sound events are evaluated with different state-of the-art methods.

For the specific case of soundscape classification, ACF and, specifically, Narrow-Band ACF (NB-ACF), have been used in the past years. For example, in Reference [25], Valero et al. put forward a classification of audio scenes using NB-ACF. In this study, NB-ACF is compared to other state-of-the-art signal features using 15 different audio scenes and achieving higher recognition rates regardless of the classifying algorithm. Later on, Jeon et al. classified urban park soundscapes in Reference [12] using ACF parameters and validating some of them as good indicators for an effective classification of the studied soundscapes.

#### 2.1.2. Machine Learning Techniques

There are many classifying algorithms that appear consistently in many works related to multi-class AED. One of the most spread choices is the Gaussian Mixture Model (GMM), which appears in conjunction with all kinds of corpora in the literature.

In the aforementioned reference [6], the features extracted are fed into classifiers based on GMM and Hidden Markov Models (HMM). In one of the experiments conducted in this study (smart-home scenario), GMM clustering proved to be the best algorithm in truly detecting abnormal audio events while keeping false detections to the minimum. Four different machine learning methods are used in Reference [26]: Discriminant Analysis (DA), SVM, GMM, and kNN, in order to identify anomalous noise events in a road traffic scenario. The study sets two different areas: suburban and urban, and evaluates their performance in terms of the macroaveraged F1 measure. kNN and SVM (followed by GMM) are the best performers in both scenarios, while DA is the worst rated algorithm in terms of F1-score.

In Reference [35], the indoor and outdoor corpora, including baby cries and smoke alarms, are classified using GMM, SVM, and Deep Neural Networks (DNN). Authors point out that GMM yields a similar Equal Error Rate (EER) or classification performance to SVM but with a much lower computational cost. In addition, in Reference [40], a binary GMM-based classifier is applied in an indoor AED and classification context. GMM is also one of the most widely chosen algorithm when classifying birds sounds and birdsongs [41,42]. In Reference [43], 165 bird syllables from up to 95 bird species are classified using GMM. Furthermore, in Reference [21], Hervás et al. model data from a synthetic dataset with GMMs in order to detect *Botaurus stellaris* (an endangered bird species).

In addition to Reference [26], kNN has been used as a classifier in other studies, as found in Reference [11]. In this case, kNN is compared to Fisher’s Linear Discriminant (FLD) using a 4-fold cross validation scheme and MFCC and GTCC as features extraction methods. kNN outperforms FLD in the first implementation of the detector but underperforms it in the second one. More recently, kNN was the preferred algorithm in Reference [13] when detecting several audio events in a forest area.

Different approaches to Neural Networks are being used as classifying methods: standard Neural Networks (NN), Deep Neural Networks (DNN), and Convolutional Neural Networks (CNN). It was already mentioned that DNN were used in Reference [35], but another relevant example can be found in Reference [27], where 36 bird species from Tonga lake were classified and recognized with the goal of monitoring their habitat.

## 3. Corpora Description

A total of five sound corpora were employed in this work, in order to widen the type of sounds and environments. In this section, the composition and origin of each corpus is described.

### 3.1. Indoor Sounds

The first corpus is a collection of indoor sounds originally compiled in Reference [44] from collaborative sound libraries [45] and subsequently pruned. The sampling rate used is 48 kHz in most of the samples and is otherwise 44.1 kHz. The total length of this corpus is 2932 s, and the total number of samples is 142. Each one of the ten classes is composed by 4 to 30 samples that present a variable length (between 0.2 and 211.3 s), as observed in Table 1.

### 3.2. Outdoor Sounds

The second corpus is composed by 15 different outdoor general sounds collected from at least four different origins for guaranteeing data variability. It is composed by audio samples collected from common sound libraries [46], collaborative sound databases [45], and manual recordings performed in real environments [47]. They are high quality loss-less (WAV) recordings obtained at a sampling rate ranging from 44.1 kHz to 48 kHz. The length of each sample is set to 4 s, and there are between 150 and 300 samples for each class, as we can see in Table 2. This corpus contains a total of 3472 samples (files). The total length of the corpus is 13,888 s (almost 4 h of duration).

### 3.3. Soundscapes

The third corpus is composed by fifteen different soundscapes, including indoor and outdoor settings. A detailed description can be found in Table 3. This corpus was first used in Reference [48]. Part of the recordings was made using a Bruel & Kjaer 2250 sound level meter (https://bksv.com/downloads/2250/be1713.pdf). They are high quality loss-less (WAV) recordings obtained at a sampling rate of 48 kHz. For diversity purposes, part of the corpus was complemented with samples extracted from Reference [45]. Each soundscape is composed by 150 to 300 samples recorded at four (or more) different locations. As in the second corpus mentioned, each sample has a 4-s length. The total duration of this corpus is 13,888 s (almost 4 h, too) distributed in 3472 samples.

### 3.4. Surveillance-Related Sounds

The fourth corpus includes six classes of environmental surveillance-related sounds. This corpus was first used in Reference [49] and is composed by sounds obtained of common sound libraries [45,46]. They are high quality loss-less (WAV) samples with a sampling rate of 44.1 kHz. Each class contains between 50–100 samples that present a variable duration (between 0.2 and 4.6 s), as we can see in Table 4. There are 470 total samples (files) in this corpus, which account for a total of 680 s.

### 3.5. Bird Sounds

The fifth and last corpus is thoroughly described in Reference [50]. It is a collection of bird sounds (call, drumming, and song) of seven *Picidae* species obtained from the Xeno-Canto repository [51]. The WAV files are sampled at 44.1 kHz. Each class contains from 49 to 146 samples that also present a variable length (between 0.2 and 10.5 s), as seen in Table 5. The original corpus also contained 523 samples from an additional class Silence, which were removed for consistency with the other datasets used. The total file count of the corpus amounts 1146 files, for a total length of 2190 s.

**Figure 1:**
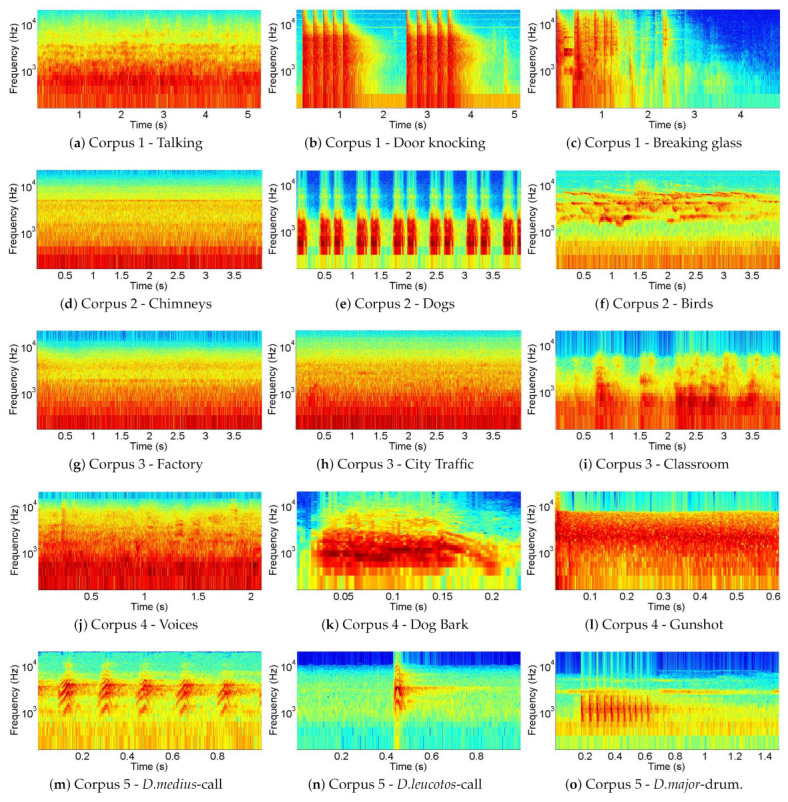
Selection of representative spectrograms of some of the classes in the *corpora*. The temporal window varies between approximately 0.2 and 5 s, but the vertical axis (Hz) is fixed.

A selection of spectrograms for some different classes of sounds contained in each corpus is shown in Figure 1. Each row corresponds to one particular corpus. The first two rows show the spectrograms of three indoor sounds (i.e., talking, door knocking, and breaking glass) and three outdoor sounds (i.e., chimneys, dog barking, and mixed birds). Talking (Figure 1a), like frying or chimneys, is quite stationary. In contrast, door knocking (Figure 1b) and breaking glass (Figure 1c) are impulsive sounds of short duration. In the case of door knocking or dog barking (Figure 1e), they are repetitive, and, in the case of breaking glass, they are usually not. Chimneys (Figure 1d) and birds (Figure 1f) have some dominant frequencies that are continuously present in time in the first case and are intermittent in the second one.

The third row shows three examples of sound scenes (i.e., factory, city traffic and classroom). The soundscapes, being formed by a combination of several sound sources, are quite unchanging in time (on a spectral level) as can be seen in (Figure 1g) and (Figure 1h) even if some discrete sounds can be spotted on occasions like in the classroom example (Figure 1i).

In the fourth row, there are three examples of surveillance-related sounds (voices, dog barks, and gunshots). Most of these sounds are impulsive (like footsteps, dog barks (Figure 1k), and gunshots). In the gunshot case, the duration of the main sound event is extremely short, as shown in Figure 1l. Finally, in the last row, three examples of the *Picidae* species contained in the fifth and last corpus can be found. While some birds have a repetitive call pattern, like the *Dendrocopos medius* (Figure 1m), other species present a single impulsive one, like the *Dendrocopos leucotos* (Figure 1n). Changes in the frequency range, periodicity, and duration can be observed between the various *Picidae* species.

## 4. Description of Selected Audio Features Extraction Methods and Machine Learning Algorithms

In this section, a brief description of the key features of the FE and ML algorithms used in this present work is provided.

### 4.1. Feature Extraction Techniques

#### 4.1.1. Mel Frequency Cepstrum Coefficents (MFCC)

MFCC [23,30] is a filter banks-based cepstral-domain features extraction method. After applying the Fast Fourier Transform (FFT) to a given windowed signal, a Mel-Scaled filter bank (based on a nonlinear frequency scale inspired by physiological evidence of the way the human perception of speech signals works) is used. This triangular filter bank divides the spectrum non-linearly following the Mel scale where the lower frequency filters have smaller bandwidth than the higher frequency ones. The Mel scale follows a linear frequency spacing below 1 kHz and a logarithmic spacing above 1 kHz. The number of filters and the chosen frequency range must be previously defined. The last stage consists of ranging the coefficients according to their significance, which is accomplished by calculating the Discrete Cosine Transform (DCT) of the logarithmic outputs from the filter bank.

#### 4.1.2. GammaTone Cepstral Coefficients (GTCC)

GTCC is another filter banks-based cepstral-domain features extraction method that was proposed by Patterson et al., in Reference [52]. The GammaTone function provides several characteristics that make these filters suitable for modeling the human auditory system’s spectral response [53,54].

The GammaTone function is calculated as the multiplication between the Gamma distribution function and a sinusoidal tone:

(1) $$gt\left(t\right)=Kt^{n-1}e^{-2\pi Bt}\cos\left(2\pi f_{c}t+\varphi \right)\;t\geq 0,$$

where *K* is an amplitude factor, *n* is the filter order, *B* is a bandwidth parameter, $f_{c}$ is the filter central frequency, and φ is the phase shift.

The duration of the filter’s impulse response is directly related to the Equal Rectangular Bandwith (ERB), which is a measure used in psychoacoustics to approximate the bandwidth of the human auditory filters in each point of the cochlea. For a given order *n*, there is a fixed relationship between *B* and the ERB; for the specific case of a 4th-order GT filter, B=1.019 times ERB.

The ERB is calculated as:

(2) $$ERB={\left[{\left(\frac{f_{c}}{EarQ}\right)}^{n}+{minBW}^{n}\right]}^{\frac{1}{n}},$$

where $f_{c}$ is the filter central frequency, EarQ is the asymptotic quality of the filter at the higher frequencies, minBW is the minimum bandwidth at the lower frequencies, and *n* is the order of the approximation.

The central frequency of each filter $f_{ci}$ can be calculated with the following equation:

(3) $$f_{ci}=\left(f_{high}+EarQ\;minBW\right)e^{-\frac{i\;step}{EarQ}}-EarQ\;minBW,$$

where $f_{high}$ refers to the higher frequency considered in the filter bank, EarQ and minBW are the ERB parameters that already appeared in Equation (2), *i* is the GT filter index, and step corresponds to the distance between the filters and can be determined with:

(4) $$step=\frac{EarQ}{N}\ln\left(\frac{f_{high}+EarQ\;minBW}{f_{low}+EarQ\;minBW}\right),$$

with *N* being the number of filters, and $f_{low}$ being the lowest frequency considered.

The process of feature extraction using the aforementioned GT filter is analogous to the one described in Section 4 replacing the Mel-Scaled filter bank for the Gammatone filter bank.

#### 4.1.3. Narrow-Band Autocorrelation Function Features (NB-ACF)

To implement this technique, the chosen signal is framed and an A-weighing filter is applied in order to model the spectral response of the human auditory system. Subsequently, the windowed signal is split into *N* narrow band signals with the help of a Gammatone filter bank, and the autocorrelation function (ACF) is calculated on each of them. Finally, the ACF is analyzed by extracting the following adapted five parameters that are merged into a single feature vector [55]:

1. $\phi_{i}\left(0\right)$: energy of the *i*-th narrow band signal (5). The vector $\phi_{i}\left(0\right)$∀i∈{1…N} represents the power spectral response of the signal.
  (5) $$\phi_{i}\left(0\right)=\frac{1}{T}\int_{0}^{T}y_{i}{\left(t\right)}^{2}dt.$$
2. $\tau_{e,i}$: effective duration of the normalized envelope of the *i*-th band ACF signal. It gives information about the reverberation component contained in this band. It is calculated as the time that the $10\log\left(\phi_{i}\left(\tau \right)\right)$ function takes to decay 10dB.
3. $\tau_{1,i}$: delay of the first peak found in the i-th band ACF signal. This parameter is related to the dominant frequency contained within the *i*-th band signal. It can be calculated as the delay of the largest $\phi_{i}\left(\tau \right)$, starting from the first zero crossing ($T_{K}$), as we can see in (6).
  (6) $$\tau_{1,i}=\arg\max_{\tau }\left\{\frac{1}{\phi_{i}\left(0\right)\left(T-T_{K}\right)}\int_{T_{K}}^{T}y_{i}\left(t\right)y_{i}\left(t+\tau \right)dt\right\}.$$
4. $\phi_{1,i}$: amplitude of the first peak found in the *i*-th band ACF signal ($\tau_{1,i}$). The vector $\phi_{1,i}$∀i∈{1…N} indicates the pitch strength at the different frequency bands. In other words, a low value of $\phi_{1,i}$ means that the dominant frequency of this band is not important within the overall signal. On the contrary, a high value of this feature represents a strong pitch in the *i*-th band. Both $\tau_{1,i}$ and $\phi_{1,i}$ are especially useful in auditory soundscapes and other scenarios where coexist different sound sources. This parameter can be computed as follows:
  (7) $$\phi_{1,i}=\max\left\{\frac{1}{\phi_{i}\left(0\right)\left(T-T_{K}\right)}\int_{T_{K}}^{T}y_{i}\left(t\right)y_{i}\left(t+\tau \right)dt\right\}.$$
5. $AZCR_{i}$: the autocorrelation zero crossing rate is the number of times that the ACF of the *i*-th band crosses the zero amplitude level (8).
  (8) $$AZCR_{i}=\frac{1}{T-1}\sum_{t=0}^{T-1}\left|sgn\left(y_{i}\left(t\right)\right)-sgn\left(y_{i}\left(t+1\right)\right)\right|.$$

### 4.2. Machine Learning Techniques

#### 4.2.1. K-Nearest Neighbors (kNN)

**Figure 2:**
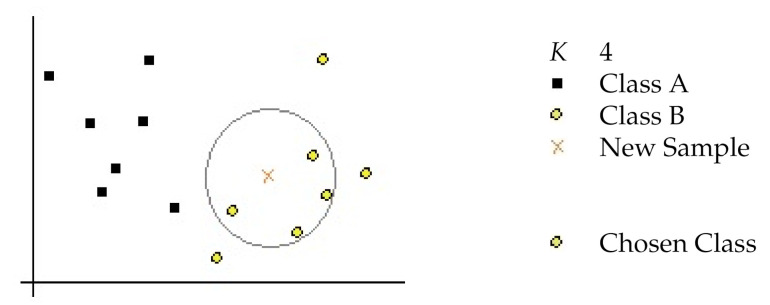
Example of *k*-Nearest Neighbor (kNN) class prediction.

kNN [56] is a widely used classification technique which has a high predictive power. It has also the advantage of being an instance-based algorithm easy to implement. The entire training dataset serves as the model for kNN. When a new data instance must be classified, the kNN algorithm will search through all the training set for the *K* instances that are deemed to be more similar following some appropriate measurement (i.e., the Euclidean distance for real-valued data), as we can see in Figure 2. After that, the algorithm summarizes the prediction attribute of these k-most similar instances and uses this to predict the class for the new observed data.

#### 4.2.2. Neural Networks (NN)

NN are designed to model the human brain behavior when performing a particular task. As such, it is a parallel distributed machine that is constituted by simple processing units (neurons) with the capability of storing knowledge. This knowledge is acquired from the system inputs through a learning process and uses synaptic weights to store it [57].

Several logistic units are structured in layers to form a neural network. There is always a first layer of input units followed by one or multiple hidden layers of neurons and by a final output layer. The activation functions are calculated using the different weights or parameters of the network.

A neural network can be effectively used as a machine learning method by using a training dataset to compute the fitting weights of each internode relationship through a backpropagation algorithm. For further understanding of the way the backpropagation algorithm works, please read Reference [57], Chapter 4.

#### 4.2.3. Gaussian Mixture Models (GMM)

A mixture model is a combination of *K* component distributions that collectively make a mixture distribution f(x) (9). In a Gaussian Mixture Model, the $f_{i}\left(x\right)$ components are chosen to be normal or Gaussian distributions.

(9) $$f\left(x\right)=\sum_{i=1}^{K}w_{i}f_{i}\left(x\right),$$

where $w_{i}$ represents a mixing weight for the *i*-th component.

The GMM is a multimodal distribution that allows a clustering of the data similar to that achieved with the k-means algorithm [58]. The goal is to model the data in the training set using a mixture of Gaussians given a number of clusters. The value of $w_{i}$ and the parameters of each Gaussian distribution are estimated using the Expectation Maximization (EM) algorithm [59].

## 5. Experiments

The goal of the following evaluations is to determine which of the pool of FE methods and ML algorithms chosen achieves better classification rates in the different evaluated datasets. To that extent, accuracy, in addition to recall, will be the main metrics used to sort out the different settings. Afterwards, the most accurate FE/ML pairing will be scrutinized via its confusion matrices in the search for the most often correctly and erroneously classified sounds.

### 5.1. Experimental Setup

During the process of feature extraction, a sampling rate of 48 kHz was used. The framing of the input signal depends on the feature extraction method selected. A 30-ms frame length is chosen when working with MFCC and GTCC with a 50% overlapping of the time frame applied (15-ms step) [47]. On the contrary, a longer time frame (500 ms) is necessary in the case of NB-ACF [60] and a 100-ms step is used in order to improve the precision of the analysis while avoiding information loss between consecutive frames (following the recommendation of Reference [61]).

To generate the features vector through the baseline MFCC algorithm, the setup in Table 6 was used. The filter bank consists in a first set of linearly-spaced overlapping filters followed by a second set of logarithmically-spaced ones. The two sets combined cover all the audible spectrum.

An optimized GTCC implementation is used, adapted from the algorithm in Reference [47] with a Glasberg & Moore ERB model [62] (EarQ=9.26 and min BW=24.7) and 48 4th-order GT filters. The lower frequency is set at 20 Hz and the higher frequency at the Nyquist limit of 24 kHz. The actual $f_{ci}$ frequencies of the filters are obtained using Equation (3).

In the NB-ACF scenario, the framed signal is passed through a filter bank that splits the signal into N=48 narrow band signals contained within 20 Hz and 24 kHz. The algorithm is an adapted version of the one proposed in Reference [25]. The five parameters described in Section 4.1.3 are subsequently calculated for each individual narrow band in order to generate the features vector.

A heuristic approach was used during the set-up of the ML algorithms. Sweeps involving several parameters were used to determine and choose the following higher-performers on a classification accuracy level. The number of neighbors from the kNN (K=3) and the number of Gaussian from the GMM (G=32) were empirically chosen in order to maximize the accuracy of the classification algorithms. The neural network used has one single hidden layer containing NHL=100 neurons. The audio patterns are divided into train and test datasets using a 4-fold cross validation scheme following previous works, such as Reference [47].

### 5.2. Experimental Results

The five corpora described were tested using three feature extraction methods (MFCC, GTCC, and NB) and three machine learning algorithms (kNN, NN, and GMM) giving a total of 45 sets of results. It is unfeasible to explicitly include all the detailed results, including confusion matrices, in this present paper due to their extension. Nevertheless, all 45 sets were evaluated to draw the final conclusions.

#### 5.2.1. Accuracy Results

In this current work on audio classification, accuracy is defined as the ratio of correctly classified data frames to the total frames attempted (10).

(10) $$Accuracy\;=\;\frac{TruePositives\;+\;TrueNegatives}{Total\;Audio\;Frames}.$$

**Figure 3:**
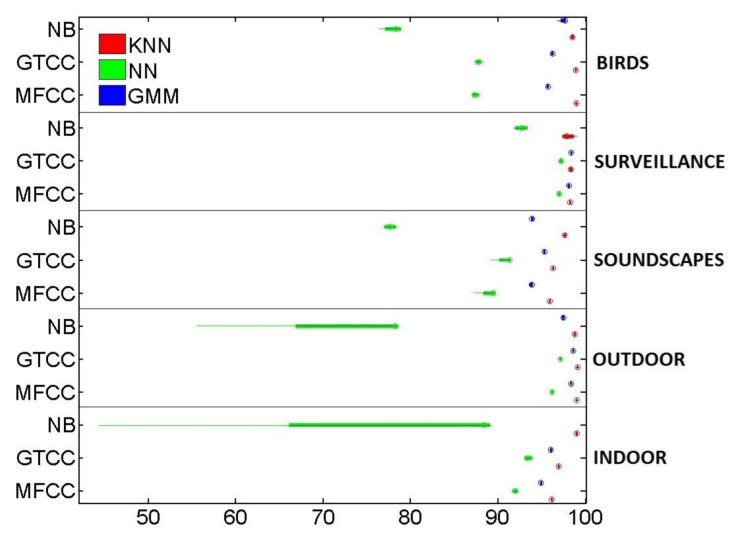
Boxplot of the detailed Accuracy’s Mean and Variance in the 4-fold simulations.

Figure 3 summarize the global accuracy’s mean (resulting of the 4-fold simulation scheme) of the studied scenarios. On the one hand, GTCC outdoes MFCC in all but one simulation, i.e., when it is used in conjunction with the kNN algorithm on the birds dataset. And, even in this exceptional case, their performance is almost identical. On the other hand, NB-ACF proves to be an optimal solution in some datasets but only when it is paired with kNN. That is especially true when it is used to classify soundscapes which is to be expected given the continuous nature of the sounds. On the contrary, it performs especially poorly when trying to categorize impulsive sounds, such as the surveillance-related ones.

When it comes to the machine learning algorithms, kNN outperforms both GMM and NN in most of the cases. Only the surveillance-related dataset gives GMM comparable or even slightly better results when combined with GTCC. GMM is second in the accuracy ranking, while NN lags behind. It is to be noted that the set GMM + NB-ACF struggles to converge when there is not enough data available in the dataset.

As we can see in Figure 3, the winning pairing is GTCC + kNN, which always gives one of the two best performances in all simulations. On the other hand, NB-ACF + NN proves to be the worst choice overall due to its larger variance and lower mean.

Some datasets’ results are more dependant on the combination of FE method and ML algorithm than others. In Figure 3, when dealing with the outdoor and surveillance datasets, similar results are achieved in most FE/ML pairings, except the aforementioned NB-ACF + NN one. Conversely, in the remaining datasets, the accuracy varies significantly more.

#### 5.2.2. Recall Results

Some other metrics, such as Recall and F1-score, can give us some more insight and validate the results presented through the previous accuracy’s analysis. Recall or sensitivity is defined as the ratio of correctly predicted positive observations (true positives) to all the observations of the current class (11).

(11) $$Recall=\frac{TruePositives}{TruePositives+FalseNegatives}.$$

**Figure 4:**
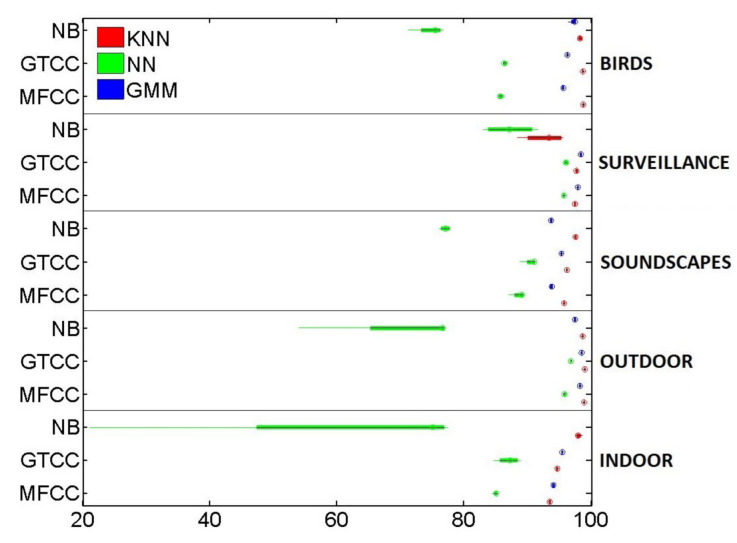
Boxplot of the Recall’s Mean and Variance in the 4-fold simulations.

Indeed, in Figure 4, even if the mean and variance of Recall in the 4-fold simulations scheme are a little bit lower and wider than the ones observed in Figure 3, the overall relative performance of the different FE/ML combinations remains almost the same, with two notable exceptions.

Firstly, in some of the datasets where kNN outperformed GMM at an accuracy level, they swap positions when Recall is taken into account. It is the case of the indoor and surveillance-related sounds. In both cases, the pairings of GMM with GTCC or MFCC offer better Recalls than the pairings of kNN with the same FE methods.

Secondly, in the surveillance-related database, all NB-ACF solutions widely underperform GTCC and MFCC on a Recall basis. Not only do they offer a below par mean figure but also a larger variance, even with the normally robust kNN. This last observation is also confirmed with another metric, F1-score, which otherwise parallels accuracy top to bottom.

#### 5.2.3. Detailed Study of the Optimum Setting

As seen in Section 5.2.1. the more accurate overall set of FE method and ML algorithm is GTCC+kNN, which consistently appears as one of the top two-tiers in each dataset both in accuracy and F1-score metrics. Moreover, this combination is also one of the top three performers when considering Recall.

**Figure 5:**
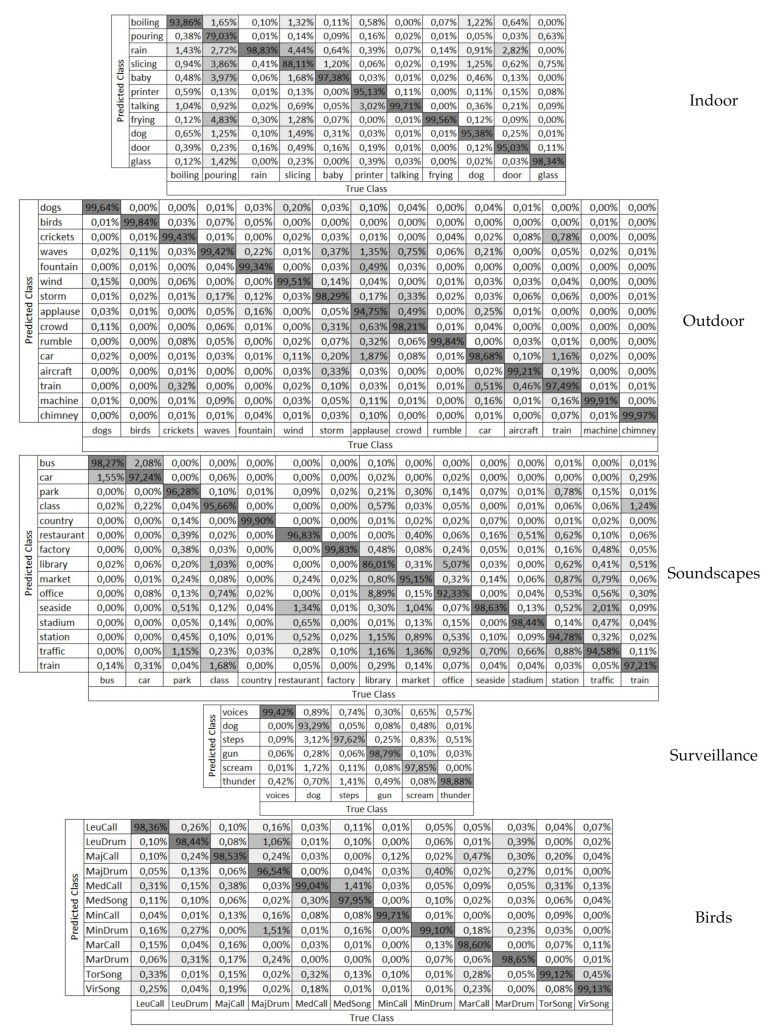
Confusion matrices of the optimum setting (Gammatone Cepstrum Coefficients (GTCC)+kNN) in the analyzed *corpora*.

Confusion matrices are shown in Figure 5, in order to ease our detection of which classes are more often missclassified and otherwise. A glimpse to these matrices show us some interesting facts. For starters, wrongly predicted classes are not evenly distributed. Thus, erroneously classified sounds are chiefly predicted into a limited set of classes. In fact, there are some examples were a predominant swap between two specific classes takes place. These phenomenon happens to a greater extent in those sounds with a lower true positives’ rate. Some examples would be slicing sounds in the indoor dataset, which are often mistakenly predicted as rain or the library and office soundscapes, which are the most frequently swapped.

**Figure 6:**
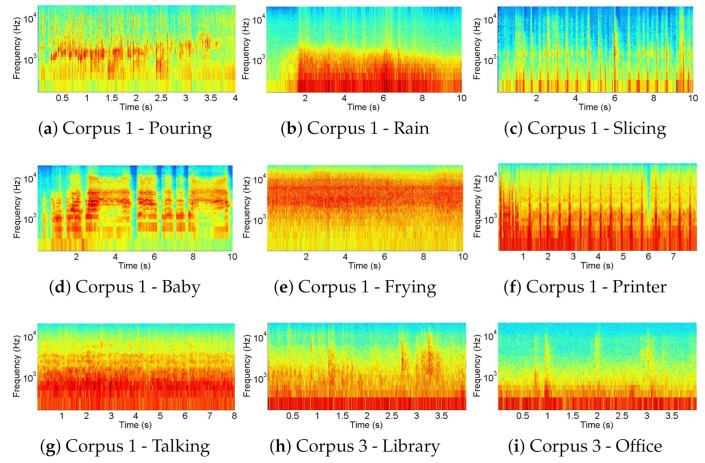
Selection of spectrograms of the most often mis-classified classes in the *corpora* for illustrative purposes. The temporal window varies between approximately 4 and 10 s, but the vertical axis (Hz) is fixed.

Most of the classes attain very high accuracy rates, no matter the database considered, except for one or two classes which are clearly below par. The classes that present higher confusion are shown in Figure 6 for illustrative purposes. Regarding the first dataset, most classes, as many as 8, are correctly classified more than 95% of the time. On the other hand, pouring and slicing sounds have an accuracy below 90%. Pouring is mainly confused with frying, slicing, and baby, but also with rain, which is a sound with a wide spectrum, despite its lack of low-frequency component. Slicing, with an 88% accuracy, also presents its main confusion with rain, which, with a visual analysis, we conclude may be confused with the impulse sounds that slicing presents. Another misclassification is between printer and talking, despite the fact that, in this case, the error is small, in about 3% of the times. As shown in Figure 6, most of the classification errors found correspond to wide spectra; some of them belonging to continuous sounds and others to temporally impulse noise, as in slicing, baby, or printer.

As for the second database, only applause has less than 95% of true positives, while up to 13 classes have over 98% accuracy. Nevertheless, this corpus present very stable results; with high accuracy values of classification and no clear deviation in the misclassification scenario, all the confusions present very low values of appearance.

In the soundscapes dataset, the situation is similar to the indoor one with 11 classes over 95% and only libraries and offices below 93%. Despite the fact that the global performance of the FE and ML in this corpus is brilliant with accuracy values mainly over 90%, there are two clear confusions, between libraries and offices, which, by the way, present the lower accuracy values. In Figure 6, it is shown that both sounds have their main energy distribution in low frequencies but have punctual temporal components of higher frequencies. This can be explained due to the high similarities between the two environments, taking into account that this corpus works with soundscapes (and not isolated events). Despite the fact that those spectra correspond to different soundscapes, they present a similar spectrogram, which leads to confusion.

Dog barking obtains the worst result in the surveillance-related dataset, with just over 93%, and only two classes fail to reach 98% between the birds, i.e., major-drum and medius-song.

Finally, some of the classes produce almost perfect classification records, with close to 100% accuracy rates, as seen in Table 7. The authors would like to state at this point that the results are concluded by means of a 4-fold cross validation algorithm, which ensures that no overfitting occurs when conducting the training and testing of the algorithms, but none of these algorithms have been tested in a real-operation environment, where those excellent accuracy results would be lowered by the diverse occurrences of the real-world unexpected situations.

## 6. Conclusions

This paper presents a detailed evaluation of three FE methods (MFCC, GTCC, and NB-ACF), together with three ML methods (kNN, NN, and GMM), tested over five acoustic corpora corresponding to diverse environments (indoor, outdoor, soundscapes, surveillance, and birdsong), to be deployed to perform real-time in a WASN framework. Despite the fact that the used algorithms have been widely studied in the literature, a wide survey, including the combination of the three FE algorithms with the three ML procedures, against the five corpora, shows an extensive picture of the strengths and weaknesses of the different approximations. The results show a moderate to high accuracy and recall results for most of the combinations of the FE and ML methods, using a 4-fold cross-validation technique to obtain the results.

The lower accuracy results are obtained by NB-ACF in situations where they have to support impulsive acoustic sounds, despite the fact that pairing with kNN presents the best possible results in soundscape detection. GTCC produces the best results in most of the corpora, outperforming MFCC, which is traditionally considered the baseline. In terms of ML algorithms, the best results are shown by kNN, apart from the surveillance-related corpus, where GMM shows similar results.

These results lead us to present a general-purpose proposal using GTCC and kNN for all the corpora class identification. A deeper analysis of this combination manifests that the results show both high accuracy and recall, as well as that there are few classes in the indoor corpus and the soundscape corpus that present relevant confusion values. Nevertheless, we have to state that there is no mean accuracy value lower than 79% in any of the tests conducted by means of the 4-fold cross-validation.

Future work is centered in testing all this test-bed over larger corpora to evaluate their generalization capability; in terms of the outdoor corpus, it will be tested over a wide suburban [29] and urban [28] corpora, gathering more than 300 h of labeled data, and collected in two 24-sensor WASNs, in order to test the GTCC+kNN proposal into the real-operation environment, not only the dataset idea. Another issue that will be studied in the future is the feasibility of the real-time implementation of the algorithm proposal depending on the computation capability of each of the nodes of the WASN because recent studies in terms of privacy and data transmission efficiency show us that the most preferred implementation [10,63] is the processing of all signals in the proper sensor when this is already deployed in the place under study. A deeper analysis of the computational load of each of the algorithms, as well as the suitability of their implementation on each type of processor in the nodes, will be analyzed.

## Figures and Tables

**Table 1:** Indoor corpus composition [44].

Category	File Count	Length (s)	Duration Distribution (s)
Breaking Glass	30	139.14	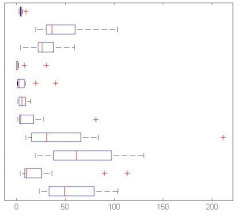
Rain	10	452.95	
Slicing	7	206.89	
Printer	17	46.87	
Door Knocking	15	101.57	
Pouring	10	59.38	
Dog Barking	15	194.88	
Frying	12	607.19	
Talking	8	542.16	
Boiling	14	355.86	
Baby Cry	4	225.53	
Total	142	2932.43	

**Table 2:** Outdoor corpus composition [47].

Category	File Count	Length (s)
Dogs	150	600
Birds	245	980
Crickets	300	1200
Sea Waves	300	1200
Fountain	242	968
Wind	158	632
Thunderstorm	256	1024
Applause	172	688
Crowd	194	776
City rumble	238	952
Car	200	800
Aircraft	182	728
Train	238	952
Machinery	297	1188
Chimneys	300	1200
**Total**	3472	13,888

**Table 3:** Soundscapes corpus composition [48].

Category	File Count	Length (s)
Inside Bus	284	1136
Inside Car	300	1200
Inside Train	236	944
Station	198	792
Classroom	200	800
Office	288	1152
Factory	250	1000
Stadium	269	1076
Restaurant	193	772
Library	173	692
City Park	200	800
City Traffic	253	1012
City Market	227	908
Countryside	150	600
Seaside	251	1004
Total	3472	13,888

**Table 4:** Surveillance-related corpus composition [49].

Category	File Count	Length (s)	Duration Distribution (s)
Thunders	65	136.5	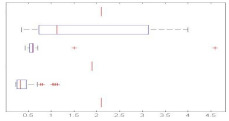
Screams	50	92.05	
Gunshots	85	57.83	
Footsteps	100	190	
Dog Barks	90	35.54	
Voices	80	168	
Total	470	679.93	

**Table 5:** Birds corpus composition [50].

Category	File Count	Length (s)	Duration Distribution (s)
*Dendrocopos major*	146	184.98	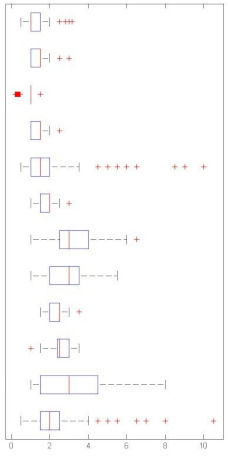
- call			
*Dryocopus martius*	140	197.5	
- call			
*Dendrocopos leucotos*	146	128.13	
- call			
*Dendrocopos major*	62	88.5	
- drumming			
*Dendrocopos minor*	105	209	
- call			
*Dendrocopos minor*	127	227.5	
- drumming			
*Jynx Torquilla*	102	324.35	
- song			
*Picus viridis*	49	142.5	
- song			
*Dendrocopos leucotos*	50	119	
- drumming			
*Dryocopus martius*	53	133	
- drumming			
*Dendrocopos medius*	42	136.91	
- song			
*Dendrocopos medius*	124	298.51	
- call			
Total	1146	2189.88	

**Table 6:** Mel Frequency Cepstrum Coefficients (MFCC) bank filter setup.

Lower Frequency	20 Hz	Linearly-spaced Filters	14
Higher Frequency	22 kHz	Log-spaced Filters	34

**Table 7:** Complete list of classes with a higher than 99.5% accuracy rate.

Dataset	Class	Accuracy	Dataset	Class	Accuracy
Indoor	Talking	99.52%	Outdoor	Dogs Barking	99.64%
Outdoor	Birds	99.84%	Outdoor	Wind	99.51%
Outdoor	Rumble	99.84%	Outdoor	Machinery	99.91%
Outdoor	Chimneys	99.97%	Soundscapes	Countryside	99.90%
Soundscapes	Factory	99.83%	Birds	Minor-Call	99.71%

## Data Availability

No new data is created or analyzed for this study.

## Abbreviations

The following abbreviations are used in this manuscript:

AAL	Ambient Assisted Living
ACF	Auto-Correlation Features
AED	Audio Event Detection
AZCR	Autocorrelation Zero Crossing Rate
CNN	Convolutional Neural Networks
DA	Discriminant Analysis
DCT	Discrete Cosine Transform
DNN	Deep Neural Networks
EER	Equal Error Rate
END	Environmental Noise Directive
ERB	Equal Rectangular Bandwidth
EU	European Union
FE	Features Extraction
FFT	Fast Fourier Transform
FLD	Fisher’s Linear Discriminant
GMM	Gaussian Mixture Model
GP-GPU	General Purpose - Graphics Processing Unit
GPS	Global Positioning System
GTCC	GammaTone Cepstrum Coefficients
HMM	Hidden Markov Models
IoT	Internet of Things
kNN	*k*-Nearest Neighbor
LLD	Low-Level Descriptors
MFCC	Mel Frequency Cepstrum Coefficients
ML	Machine Learning
NB	Narrow Band
NB-ACF	Narrow Band Auto-Correlation Features
NN	Neural Networks
PWP	Perceptual Wavelet Packets
RTN	Road Traffic Noise
SVM	Support Vector Machines
WASN	Wireless Acoustic Sensor Network

## References

[1] Davies A.C., Velastin S.A. (2005). A progress review of intelligent CCTV surveillance systems. Proc. IEEE IDAACS.

[2] Schwartz A. (2012). Chicago’s video surveillance cameras: A pervasive and poorly regulated threat to our privacy. Northwest. J. Technol. Intell. Prop..

[3] Alías F., Alsina-Pagès R.M. (2019). Review of Wireless Acoustic Sensor Networks for Environmental Noise Monitoring in Smart Cities. J. Sens..

[4] Wang W., Seraj F., Meratnia N., Havinga P. Privacy-aware environmental sound classification for indoor human activity recognition. Proceedings of the PETRA ’19: 12th ACM International Conference on PErvasive Technologies Related to Assistive Environments.

[5] Vafeiadis A., Votis K., Giakoumis D., Tzovaras D., Chen L., Hamzaoui R. (2020). Audio content analysis for unobtrusive event detection in smart homes. Eng. Appl. Artif. Intell..

[6] Ntalampiras S., Potamitis I., Fakotakis N. (2011). Probabilistic Novelty Detection for Acoustic Surveillance Under Real-World Conditions. IEEE Trans. Multimed..

[7] Vacher M., Portet F., Fleury A., Noury N. Challenges in the processing of audio channels for ambient assisted living. Proceedings of the 12th IEEE International Conference on e-Health Networking, Applications and Services.

[8] Rashidi P., Mihailidis A. (2012). A survey on ambient-assisted living tools for older adults. IEEE J. Biomed. Health Inform..

[9] Bouakaz S., Vacher M., Bobillier Chaumon M., Aman F., Bekkadja S., Portet F., Guillou E., Rossato S., Desserée E., Traineau P. (2014). CIRDO: Smart companion for helping elderly to live at home for longer. IRBM.

[10] Alsina-Pagès R., Navarro J., Alías F., Hervás M. (2017). HomeSound: Real-Time Audio Event Detection Based on High Performance Computing for Behaviour and Surveillance Remote Monitoring. Sensors.

[11] Socoró J., Ribera G., Sevillano X., Alías F. Development of an Anomalous Noise Event Detection Algorithm for dynamic road traffic noise mapping. Proceedings of the 22nd International Congress on Sound and Vibration (ICSV22).

[12] Jeon J., Hong J. (2015). Classification of urban park soundscapes through perceptions of the acoustical environments. Landsc. Urban Plan..

[13] Chaudhary M., Prakash V., Kumari N. Identification Vehicle Movement Detection in Forest Area using MFCC and KNN. Proceedings of the 2018 International Conference on System Modeling & Advancement in Research Trends (SMART).

[14] Sevillano X., Socoró J., Alías F., Bellucci P., Peruzzi L., Simone R., Coppi P., Nencini L., Cerniglia A., Bisceglie A. (2016). DYNAMAP—Development of low cost sensors networks for real time noise mapping. Noise Mapp..

[15] Mydlarz C., Salamon J., Bello J. (2017). The implementation of low-cost urban acoustic monitoring devices. Appl. Acoust..

[16] Jati A., Nadarajan A., Mundnich K., Narayanan S. Characterizing dynamically varying acoustic scenes from egocentric audio recordings in workplace setting. Proceedings of the IEEE International Conference on Acoustics, Speech and Signal Processing (ICASSP).

[17] Chu S., Narayanan S., Kuo C., Mataric M. Where am i? Scene recognition for mobile robots using audio features. Proceedings of the IEEE International Conference on Multimedia and Expo, ICME.

[18] Ozkan Y., Barkana B. Forensic Audio Analysis and Event Recognition for Smart Surveillance Systems. Proceedings of the 2019 IEEE International Symposium on Technologies for Homeland Security (HST).

[19] Porter J.H., Nagy E., Kratz T.K., Hanson P., Collins S.L., Arzberger P. (2009). New eyes on the world: Advanced sensors for ecology. BioScience.

[20] Stowell D., Wood M., Stylianou Y., Glotin H. Bird detection in audio: A survey and a challenge. Proceedings of the 2016 IEEE 26th International Workshop on Machine Learning for Signal Processing (MLSP).

[21] Hervás M., Alsina-Pagès R., Alías F., Salvador M. (2017). An FPGA-Based WASN for Remote Real-Time Monitoring of Endangered Species: A Case Study on the Birdsong Recognition of *Botaurus stellaris*. Sensors.

[22] Somervuo P., Harma A., Fagerlund S. (2006). Parametric representations of bird sounds for automatic species recognition. IEEE Trans. Audio Speech Lang. Process..

[23] Mermelstein P., Chen C.H. (1976). Distance measures for speech recognition, psychological and instrumental. Pattern Recognition and Artificial Intelligence.

[24] Agrawal D., Sailor H., Soni M., Patil H. Novel TEO-based Gammatone features for environmental sound classification. Proceedings of the European Signal Processing Conf. (EUSIPCO).

[25] Valero X., Alías F. Classification of audio scenes using Narrow-Band Autocorrelation features. Proceedings of the 20th European Signal Processing Conference (EUSIPCO).

[26] Socoró J., Alías F., Alsina-Pagès R. (2017). An Anomalous Noise Events Detector for Dynamic Road Traffic Noise Mapping in Real-Life Urban and Suburban Environments. Sensors.

[27] Boulmaiz A., Messadeg D., Doghmane N., Taleb-Ahmed A. (2016). Robust acoustic bird recognition for habitat monitoring with wireless sensor networks. Int. J. Speech Technol..

[28] Alías F., Socoró J.C., Orga F., Alsina-Pagès R.M. Characterization of A WASN-Based Urban Acoustic Dataset for the Dynamic Mapping of Road Traffic Noise. Proceedings of the 6th ECSA—Electronic Conference on Sensors and Applications.

[29] Alsina-Pagès R.M., Orga F., Alías F., Socoró J.C. (2019). A WASN-Based Suburban Dataset for Anomalous Noise Event Detection on Dynamic Road-Traffic Noise Mapping. Sensors.

[30] Davis S., Mermelstein P. (1980). Comparison of Parametric Representations for Monosyllabic Word Recognition in Continuously Spoken Sentences. IEEE Trans. Acoust. Speech Signal. Process..

[31] Aurino F., Folla M., Gargiulo F., Moscato V., Picariello A., Sansone C. One-Class SVM Based Approach for Detecting Anomalous Audio Events. Proceedings of the 2014 International Conference on Intelligent Networking and Collaborative Systems.

[32] Mesaros A., Heittola T., Eronen A., Virtanen T. Acoustic event detection in real life recordings. Proceedings of the 18th European Signal Processing Conference.

[33] Salamon J., Jacoby C., Bello J. A Dataset and Taxonomy for Urban Sound Research. Proceedings of the 22nd ACM International Conference on Multimedia.

[34] Ntalampiras S. (2014). Universal background modeling for acoustic surveillance of urban traffic. Digit. Signal Process..

[35] Sigtia S., Stark A., Krstulović S., Plumbley M. (2016). Automatic environmental sound recognition: Performance versus computational cost. IEEE/ACM Trans. Audio Speech Lang. Process..

[36] Stattner E., Hunel P., Vidot N., Collard M. Acoustic scheme to count bird songs with wireless sensor networks. Proceedings of the 2011 IEEE International Symposium onWorld ofWireless, Mobile and Multimedia Networks (WoWMoM).

[37] Ventura T., de Oliveira A., Ganchev T., de Figueiredo J., Jahn O., Marques M., Schuchmann K. (2015). Audio parameterization with robust frame selection for improved bird identification. Expert Syst. Appl..

[38] Vidaña-Vila E., Navarro J., Alsina-Pagès R., Ramírez Á. (2020). A two-stage approach to automatically detect and classify woodpecker (Fam. Picidae) sounds. Appl. Acoust..

[39] Mulimani M., Koolagudi S. Locality-constrained Linear Coding based Fused Visual Features for Robust Acoustic Event Classification. Proceedings of the Interspeech 2019.

[40] Aguilar-Ortega M., Mohíno-Erranz I., Utrilla-Manso M., García-Gómez J., Gil-Pita R., Rosa-Zurera M. Multi-microphone acoustic events detection and classification for indoor monitoring. Proceedings of the 2019 Signal Processing: Algorithms, Architectures, Arrangements, and Applications (SPA).

[41] Henriquez P., Alonso J., Ferrer M., Travieso C. (2014). Review of automatic fault diagnosis systems using audio and vibration signals. IEEE Trans. Syst. Man Cybern. Syst..

[42] Ganchev T., Jahn O., Marques M., de Figueiredo J., Schuchmann K. (2015). Automated acoustic detection of *Vanellus chilensis lampronotus*. Expert Syst. Appl..

[43] Jančovič P., Köküer M. (2011). Automatic detection and recognition of tonal bird sounds in noisy environments. EURASIP J. Adv. Signal Process..

[44] Casals E. (2016). Programació Paral.lela en Processadors Gràfics Per a La Separació de Fonts Sonores en L`Entorn de La Llar. La Salle. Master’s Thesis.

[45] Collaborative (2017). The Freesound Project. https://freesound.org/.

[46] BBC (2015). The BBC Sound Effects Library: Original Series. https://www.sound-ideas.com/Product/152/BBC-Sound-Effects-Library-Original-Series.

[47] Valero X., Alías F. (2012). Gammatone Cepstral Coefficients: Biologically Inspired Features for Non-Speech Audio Classification. IEEE Trans. Multimed..

[48] Valero X., Alías F. Análisis de la señal acústica mediante coeficientes cepstrales bio-inspirados y su aplicación al reconocimiento de paisajes sonoros (spanish). Proceedings of the ACUSTICA.

[49] Valero X., Alías F. Gammatone Wavelet features for Sound Classification in Surveillance Applications. Proceedings of the 20th European Signal Processing Conference (EUSIPCO).

[50] Vidañ a Vila E., Navarro J., Alsina-Pagès R. (2017). Towards Automatic Bird Detection: An Annotated and Segmented Acoustic Dataset of Seven *Picidae* species. Data.

[51] Foundation X.C. (2017). Xeno-Canto: Sharing Bird Sounds from around the World. https://www.xeno-canto.org/.

[52] Patterson R., Moore B. (1986). Auditory filters and excitation patterns as representations of frequency resolution. Frequency Selectivity in Hear-Ing.

[53] Patterson R., Nimmo-Smith I., Holdsworth J., Rice P. An Efficient Auditory Filterbank Based on the Gammatone Function. Proceedings of the IOC Speech Group on Auditory Modelling.

[54] Patterson R., Holdsworth J., Ainsworth W.A. (1996). A functional model of neural activity patterns and auditory images. Advances in Speech, Hearing and Language Processing.

[55] Valero X., Alías F. (2013). Narrow-band autocorrelation function features for the automatic recognition of acoustic environments. J. Acoust. Soc. Am..

[56] Cover T., Hart P. (1967). Nearest neighbor pattern classification. IEEE Trans. Inf. Theory.

[57] Haykin S. (1993). Neural Networks and Learning Machines.

[58] Jaakkola T., Singh R., Mohammad A. (2006). 6.867 Machine Learning. Fall 2006. Massachusetts Institute of Technology: MIT OpenCourseWare. https://ocw.mit.edu.

[59] Bilmes J. (1998). A Gentle Tutorial of the EM Algorithm and its Application to Parameter Estimation for Gaussian Mixture and Hidden Markov Models.

[60] Fuiji K., Soeta Y., Ando Y. (2001). Acoustical properties of aircraft noise measured by temporal and spatial factors. J. Sound Vib..

[61] Valero X., Alías F., Kephalopoulos S., Paviotti M. Pattern recognition and separation of road noise sources by means of ACF, MFCC and probability density estimation. Proceedings of the Euronoise Conference.

[62] Moore B., Glasberg B. (1996). A revision of Zwicker’s loudness model. Acta Acust..

[63] Navarro J., Vidañ a-Vila E., Alsina-Pagès R.M., Hervás M. (2018). Real-Time Distributed architecture for remote acoustic elderly monitoring in Residential-Scale ambient assisted living scenarios. Sensors.

